# From Antidepressant Tianeptine to Street Drug ZaZa: A Narrative Review

**DOI:** 10.7759/cureus.40688

**Published:** 2023-06-20

**Authors:** Morgan L Wagner, Joseph Pergolizzi, Jo Ann K LeQuang, Frank Breve, Giustino Varrassi

**Affiliations:** 1 Entrepreneur Program, NEMA Research, Inc., Naples, USA; 2 Operations, NEMA Research, Inc, Naples, USA; 3 Operations, NEMA Research, Inc., Naples, USA; 4 Pharmacy, Temple University, Philadelphia, USA; 5 Pain Management, Paolo Procacci Foundation, Rome, ITA

**Keywords:** overdose, antidepressant, gas station heroin, street drug, tianna red, zaza, tianeptine

## Abstract

Tianeptine is often incorrectly described as a selective serotonin reuptake inhibitor, but it actually is a µ-opioid receptor agonist with anxiolytic effects. It has been approved since the last 1980s in about 24 countries as a treatment for depression, but it was never cleared to market in the United States for this purpose. Nevertheless, tianeptine joined the billion-dollar US market of nootropics as ZaZa or Tianna Red and is widely available online and in small shops without a prescription, to the point that it has been nicknamed “gas station heroin.” While the therapeutic dose range is about 25 to 50 mg/day, tianeptine abusers may take 100 times that amount. Tolerance occurs rapidly and users who seek to recapture the short-lived euphoric effects of the drug have to take more and more. Social media has peer-support sites for those trying to discontinue tianeptine. Tianeptine is associated with multiple side effects at high doses along with dependence, withdrawal symptoms, toxicity, respiratory depression, and even mortality. Agitation is more often a presenting symptom of withdrawal than toxicity. Tianeptine is often used by polysubstance drug abusers who may be unaware of the drug’s dangers. Few clinicians are aware of tianeptine and most urine assays do not screen for it. Greater awareness is needed for this drug and steps must be taken as tianeptine or “gas station heroin” is emerging as a new public health threat.

## Introduction and background

The atypical antidepressant tianeptine first drew considerable medical attention with its release in 1989, because its effectiveness challenges the prevailing monoaminergic hypothesis of depression [1]. Until that time, depression was pharmacologically managed by monoamine oxidase inhibitors and tricyclic antidepressants, based on the hypothesis that depression is the result of an imbalance among serotonin, noradrenaline, and dopamine. Tianeptine opened the door to a more nuanced understanding of depression, that is, neuroplasticity and a complex interplay among neurotransmitters play a role. [2]

Approved for use in about 24 countries, excluding the United States, tianeptine has been described, somewhat erroneously, as a selective serotonin reuptake inhibitor (SSRI) but accurately as an agent with an anxiolytic effect and an affinity for µ-opioid receptors [3]. Tianeptine is not cleared to the US market as a prescription drug, but it is available as a nootropic agent, that is, a supplement intended to boost mood or improve cognition. Tianeptine, sold over the counter under the brands “ZaZa” and “Tianna Red,” has found its way into the stream of drugs of abuse. Besides widespread availability in convenience stores, smoke shops, and gas stations, to the point that it has been nicknamed “gas station heroin,” tianeptine may be purchased online with few restrictions [4]. Only a few states in the United States have regulated or banned tianeptine.

First responders, emergency department staff, and clinicians in the United States may be called upon to treat tianeptine toxicity, although not all healthcare professionals are aware of its existence. Patients, too, may take tianeptine, thinking it is safe for use outside of medical supervision. 

The objective of this narrative review is to describe the potential dangers of this “old” but new street drug in the United States and elsewhere, while at the same time being cognizant of its legitimate role in the antidepressant armamentarium for prescribed use under clinical supervision.

Methods

The authors searched the PubMed database for “tianeptine” and retrieved 614 results. “Tianeptine abuse” was also searched, yielding 73 results, all of which overlapped with the first search. We searched PubMed for “ZaZa” and “Tianna Red” and obtained four and 250 results, respectively, none of which were relevant. The authors also conducted online searches using Google and Brave search engines, because there is information about the dangers and recreational use of tianeptine in news media online. “ZaZa” was not a viable search term as it yielded 6.8 million results, many of them resulting in musical references. Searching for “tianeptine” on Google yielded about 10% of those results or 685,000. The Brave search engine yielded similar results but did not state the total number of sites retrieved. No delimiters were used in the search.

Articles were included if they dealt with tianeptine abuse, misuse, withdrawal symptoms, its psychoactive effects, and tolerance. We included case studies, commentaries, news stories, and editorials. We excluded animal studies, studies about therapeutic indications of tianeptine, articles describing the role of tianeptine as an antidepressant or in other mental health conditions, new formulations of prescription tianeptine, and articles not in the English language. We included articles to help describe the pharmacological mechanisms of action of tianeptine, but this was not a main focus of this review. This yielded 38 peer-reviewed articles, plus a few relevant newspaper articles and two notices on the Food and Drug Administration (FDA) website. This was intended as a short narrative review of tianeptine in light of its use as a dangerous and potentially addictive nootropic in the United States and is not a narrative review of tianeptine overall.

## Review

Based on its chemical structure, tianeptine was originally classified as a tricyclic antidepressant, but because it did not inhibit monoamine reuptake, this description is inaccurate, although it is still used [5]. In fact, tianeptine has no effect on serotonin reuptake, but it could more accurately be described as a glutamatergic modulator with anxiolytic properties, shown to be effective in managing somatic symptoms [6]. The recent discovery that tianeptine induced neuroplastic changes has challenged the old paradigm of depression solely as a disorder of dysregulated neurotransmitters [5]. Radioligand binding studies have confirmed that tianeptine is actually a µ-opioid receptor and δ-receptor agonist; its effects can be reversed effectively with naloxone [7,8].

The therapeutic dose range of tianeptine is 25 to 50 mg/day, but doses as low as 12.5 mg/day may be effective for some patients [9]. Tianeptine is characterized by high bioavailability, limited distribution in the body, and rapid elimination [9]. Tianeptine is indicated and approved for prescription in many nations to treat depression [9,10], and there are reports of its effective use off-label to manage the symptoms of alcohol withdrawal [2]. Reported side effects of tianeptine taken as indicated and at therapeutic doses include nausea, constipation, dry mouth, drowsiness, postural hypotension, abdominal pain, headache, dizziness, and disordered dreaming [9,11].

Tianeptine misuse and abuse

Tianeptine has been described as a safe, effective, well-tolerated antidepressant prescription agent with a relatively low potential for abuse [12]. However, for individuals seeking the psychoactive effects of a drug, supratherapeutic doses of tianeptine can produce a sense of euphoria, and tolerance builds up quickly [13], such that users intending to reexperience that pleasurable sensation rapidly increase doses [14]. A series of 18 case studies of tianeptine toxicity found individuals taking doses over 100 times greater than the therapeutic dose range [3]. Very high doses of tianeptine increase the number and severity of side effects, but withdrawal symptoms set in rapidly as the drug wears off, causing users to seek more tianeptine [15]. A somewhat different but often reported abuse trajectory for tianeptine involves people with other substance use disorders, such as opioid use disorder, who try to self-manage their withdrawal symptoms using tianeptine [15].

The original idea was that in places where tianeptine was a medically recognized antidepressant and available by prescription only, this abuse potential would be blunted. In a study of 410,000 patients in France, where tianeptine has been cleared to market as an antidepressant, it was found to be the only antidepressant for which certain patients would “doctor shop,” suggesting its abuse potential [16]. In Turkey, tianeptine is available without prescription, but in its neighboring country Georgia, tianeptine is highly regulated, making it essentially unavailable. Thus, Turkey has nicknamed tianeptine “the Georgian drug,” because large numbers of Georgians travel to Turkey to obtain and even stockpile tianeptine [14]. In Russian republics, tianeptine was either scheduled as a controlled substance or removed from the marketplace entirely in 2010 and 2011 [17].

In the United States, tianeptine has found its market niche as a part of the billion-dollar nootropic supplement industry [18]. Sold under such names as “Za Za,” Pegasus, or Tianna and described as “gas station dope” or “gas station heroin,” tianeptine can easily be purchased over the counter in many parts of the United States [19]. Displayed openly on the counter in convenience stores, nutrition stores, smoke shops, and gas stations, tianeptine is available in a bottle of 15 pills that sells for around US$25 [19]. In states where tianeptine sales are more regulated, these bottles may be kept under the counter and sold by request [19]. Tianeptine is available without a prescription on a number of online sites, where it is often sold without any warnings as a supplement to improve mental acuity [20]. One of the most prominent online sources is the Tianeptine Supply Company, which promises a certificate of analysis, money-back guarantee, and expedited shipping and offers 10 g of various powder formulations for prices of approximately US$140 to US$900 but provides no information about how or why to use the product [21].

In a study of recreational tianeptine users who experienced and survived toxicity, it was found that most took tianeptine as a part of a polysubstance cocktail, sometimes with the intention of mitigating or “smoothing out” the effects of other drugs or alcohol [3]. An interesting study of social media posts related to tianeptine analyzed 210 Reddit posts made between 2012 and 2020 and found that polysubstance use was so ubiquitous that it was nicknamed a “stack,” that is, using tianeptine plus at least one other, if not several drugs [15]. Among the Reddit posts in this study, the most frequently reported reasons for taking tianeptine were to improve mood or mental sharpness, to self-medicate for pain or a mental health condition, and to manage withdrawal symptoms from other drugs [15].

Tianeptine toxicity

Reports of tianeptine toxicity made from 2000 to 2017 to poison control centers often involve polysubstance abuse, so it is challenging to determine the specific effects of tianeptine versus the other substances and their potential interactions. Of 114 tianeptine-only exposures reporting only toxicity and not withdrawal-related symptoms, 48% had neurologic, 33% cardiovascular, and 11% gastrointestinal symptoms. Of those who reported tianeptine toxicity, 24% were admitted to a critical care unit. No deaths were reported from this study [21].

Tianeptine withdrawal symptoms

Anonymous online forums provide an opportunity for individuals taking illicit substances to obtain advice, peer support, information, and encouragement from others struggling with addiction. Another Reddit forum named “Quitting Tianeptine:” has over 4,100 members who are self-described as “currently addicted.” While there is no qualitative analysis of these posts, the challenges of tianeptine withdrawal appear to be formidable and overwhelming for some respondents [22]. In a study of 24 patients undergoing medically supervised tianeptine detoxification, withdrawal symptoms included chills and trembling, sweating, muscle pain, anxiety, and depression [23]. Agitation is more frequently a presenting symptom for tianeptine withdrawal than tianeptine toxicity [24].

Protracted withdrawal symptoms have been reported with tianeptine even after an individual has completely stopped taking the drug [4]. Protracted withdrawal symptoms, which have been reported with other drugs, such as benzodiazepines, and they can occur days, weeks, months, and even years after the drug has been fully discontinued [25,26] and differ mechanistically from the acute symptoms that occur in the immediate period when the drug is discontinued. Acute withdrawal occurs as the drug is cleared from the body, but protracted symptoms are likely due to neuroplastic changes in the brain that occur during active use and resolve only very slowly after the drug has been fully stopped [27].

Case studies report on the use of buprenorphine to manage tianeptine withdrawal [28]. In one study, a 33-year-old man tapered off a daily dose of 10 mg of tianeptine over 14 days using low doses of sublingual buprenorphine (0.25 to 12 mg) in the first seven days of the taper. He was able to stop tianeptine completely with no major withdrawal symptoms or depression [29]. Note that there is no official guidance on how to taper safely off tianeptine or how to manage prolonged symptoms, if any, after the drug is discontinued.

Clinical implications

Emergency healthcare professionals and other clinicians all over the country have had to deal with a sharply increased inflow of patients who are agitated or combative under the influence of drugs, require rescue from overdose toxicity, or are in acute withdrawal [24]. Treating such patients can be challenging, because they may not be willing and/or able to reliably explain what drugs they had taken. Because tianeptine or ZaZa is marketed as a dietary supplement, otherwise forthcoming patients in distress may not think to report taking tianeptine, because they may not see a connection to their current symptoms [19]. Tianeptine can be reversed with naloxone, but because it is often used as a part of a polysubstance “stack” that includes opioids, those in drug-induced respiratory depression may be rescued successfully without tianeptine coming to the attention of the clinical team or first responders [7,8]. While it is possible to test for tianeptine in a urine assay, few drug tests include tianeptine, mainly because it is not a prescription drug and its status as a potential drug of abuse is not well known [8]. In fact, some recreational drug users tout the lack of testing for tianeptine as an advantage for taking this drug, which can go undetected at routine drug testing [19].

A statewide poison control study evaluated telephone calls taken from January 1, 2015 to March 15, 2020 for inquiries related to tianeptine [24]. In this time period, the centers received 84 calls in total related to atypical antidepressants, of which 48 involved tianeptine [24]. It is interesting to note that 77% of all calls specific to tianeptine occurred after May 2019. Twenty-seven of these tianeptine calls (56%) required a medical admission, and of those, 17 individuals were admitted to the intensive care unit [24]. The use of tianeptine varies markedly state by state. In New York, a study of poison control center calls from 2000 to 2017 found only nine reports relating to tianeptine use, five of whom reported taking the drug recreationally [30]. The Centers for Disease Control and Prevention in Atlanta has reported a dramatic increase in tianeptine exposures since 2014 and considered the drug an emerging public health risk [31]. The state of Michigan banned tianeptine in 2018 [32] with Alabama following suit in March 2021 [33]. Moreover, Ohio has issued an emergency ban on the drug in December 2022 [34]. Although other American states, such as Georgia and Tennessee, have also banned tianeptine [32], these restriction bans do not limit the availability of the drug online and do not prohibit citizens from crossing state lines to buy “gas station heroin.”

Case reports in the literature describe drug dependence [3,4,35], abuse and misuse [14,36,37,38] and even tianeptine psychosis [39]. The medical use of tianeptine under clinical supervision has been reported to result in a patient abusing the drug by escalating doses [40]. An interesting case study emerged in the United States when a chronic pain patient was denied effective analgesic prescription by his physician and sought relief in over-the-counter tianeptine only to develop tianeptine use disorder [18]. Tianeptine overdose fatalities have been reported [41], and one case report describes a 26-year-old man who used tianeptine to complete suicide [42].

While tianeptine under clinical supervision has been used in pregnancy, a case of neonatal abstinence syndrome was reported when a mother taking over 650 mg/day of tianeptine delivered a baby who appeared to be in drug withdrawal. Although both mother and infant tested negative for morphine, they were given morphine, which effectively relieved their symptoms. Thus, neonatal abstinence syndrome may occur with tianeptine and presents in a similar fashion to opioid abstinence syndrome in newborns [43].

The FDA has issued a warning about the potentially dangerous side effects of tianeptine [44] with a more vigorously worded update in 2022 [45]. Among the adverse effects described are agitation, confusion, drowsiness, sweating, rapid heart rate, hypertension, nausea, vomiting, respiratory depression, and coma. The FDA also cautions that tianeptine is potentially fatal.

Discussion

ZaZa and tianeptine in various guises are widely marketed in the United States as over-the-counter nootropics and are likely perceived by the public to be no more harmful than other over-the-counter products [19]. The course from the initial dose to dangerously high supratherapeutic doses can occur very rapidly because tolerance builds up quickly [5], and tianeptine has a very short duration of action followed by uncomfortable withdrawal symptoms [19].

The risk factors specific for tianeptine abuse are not well known. Cognitive enhancement may be the reason some take tianeptine because they are unaware of its psychoactive effects and abuse potential. Chronic pain patients who are frustrated that they can no longer obtain effective pain medications from their physicians may learn by word of mouth that “gas station heroin” or ZaZa is a potent pain reliever. Those with various substance use disorders may take tianeptine as a way to self-medicate through withdrawal symptoms, believing it to be a less dangerous substance than the substance they are discontinuing [19]. It is likely that only a very few of these individuals likely understand the dangers of taking tianeptine at high doses, and dose escalation can occur quickly. In fact, some tianeptine abusers have reached such high doses that they get tired of taking so many pills and attempt to use the drug intravenously, exposing them to further risks of infection [14].

One of the problems with tianeptine use and abuse in the United States is that the nation is flooded with all sorts of supplements and other nootropic products, such that a very few people seriously investigate a benign-looking over-the-counter drug with a silly name like ZaZa. Furthermore, many clinicians are not aware of tianeptine and fail to warn patients about the risks of this product. Finally, the abuse of tianeptine points to the fact that there are some serious and unmet medical needs in the United States, which, regrettably, ZaZa seems to address. Some people take ZaZa or related products because they suffer moderate to severe chronic pain and are unable to get the opioid analgesics that were once available to them. This means that instead of taking pharmaceutical-grade prescribed products under a physician’s guidance, these individuals have to find “something else,” which in this case is a very dangerous drug that, ironically, is not even a very good analgesic because of its short duration of action.

Another category of ZaZa users is an individual who wants to withdraw from other substances, such as oxycontin or fentanyl, but has no access to a rehabilitation center or clinical team that can help supervise the process. Some people with substance use disorder may even be fearful of asking help from physicians for discontinuation of illegal substances, because they have not had good experiences dealing with the medical establishment. Others trying to kick a drug habit may have no insurance or very limited insurance coverage, so they cannot afford long-term medical treatment. Some, even those with insurance, may live in a place where drug rehabilitation services are not available within a reasonable distance. Their use of tianeptine in this setting points to a desperate attempt to get off drugs the best way they can with minimal distress and suffering from withdrawal. There is a paucity of data on these patients and no high-quality evidence, but it seems plausible that many of them take tianeptine to get off another drug, such as an opioid or cocaine, which might result in adding a new addiction rather than resolving an ongoing addiction.

Tianeptine can be reversed with naloxone, which suggests that it is more closely related to an opioid than a classic SSRI, yet it is largely unregulated and readily accessible, even by minors. This points to a failure of regulatory systems and a failure of public health authorities to recognize and mitigate this threat.

The fact that many nations have successfully dispensed tianeptine to patients with depression with good results indicates that for users who do not seek psychoactive effects or have no history of substance use disorder, the drug may be safely used under medical supervision. However, the doctor shopping index in France suggests that among antidepressants, tianeptine has the greatest potential for abuse [16]. The abuse potential of tianeptine may reside partly in the drug and partly in the user; that is, users who seek euphoric effects for whatever reason and who are drawn to tianeptine can easily become dependent on it with such a marked tolerance that they must take very high doses to maintain the “high” they seek.

It would be beneficial if tianeptine were to be scheduled as a controlled substance and steps taken to remove it from the over-the-counter and online markets. Clinicians should actively warn patients about ZaZa or tianeptine and refer patients struggling with this drug to specialists in addiction management. The use of tianeptine is ramping up, and there is no need to add yet another dangerous substance to the supply of dangerous recreational drugs in the United States.

This narrative review has several limitations. It is based partly on scientific, peer-reviewed literature but partly on journalistic sources, news articles, websites, and social media because of the nature of the subject. The experiences of individuals with tianeptine have been published in case reports, but there are no randomized clinical trials involving subjects who take tianeptine for recreational or non-indicated reasons.

## Conclusions

The confusion surrounding tianeptine must be cleared up: it is not a nootropic or harmless drug that can safely be sold over the counter or online without a prescription. The drug offers an initial and highly pleasurable euphoria, and this psychoactive effect makes it appealing to drug abusers who may combine it with other drugs in a “stack” for polysubstance abuse. Dependency is the expected result of prolonged exposure to supratherapeutic doses of tianeptine, triggering withdrawal symptoms if the drug is stopped abruptly or drastically reduced. Greater awareness is needed by the public who see this drug merely as a harmless over-the-counter cognitive enhancer; greater awareness is needed by merchants who sell this drug believing it is benign; and greater awareness is needed among clinicians in terms of discussing this drug with patients. Guidance as to how to treat tianeptine toxicity and rehabilitation are needed as well. Tianeptine represents a new and slow-moving public health crisis that must be efficiently and immediately addressed.
